# Waking up “We” or “I”? How Start Temporal Landmarks Influence Arousal Product Preferences

**DOI:** 10.3390/bs13010067

**Published:** 2023-01-12

**Authors:** Shichang Liang, Bin Lan, Rulan Li, Min Zhang, Yuxuan Chu, Lili Teng, Lingling He

**Affiliations:** 1 School of Business, Guangxi University, Nanning 530004, China; 2 Financial Research Center, Guangxi University, Nanning 530004, China; 3 College of Economic and Management, Nanning Normal University, Nanning 530001, China

**Keywords:** start temporal landmarks, psychological resources, self-construal, arousal product preferences, arousal theory

## Abstract

Start temporal landmark is the beginning of a period of time. Previous research has established that individuals have the need for arousal at the start temporal landmarks but less research has focused on individual differences and the relationship between self and others (independent vs. interdependent). This research examines the influence of individuals’ self-construal on the relationship between start temporal landmarks and arousal product preference. Three experiments with 1136 participants were recruited from a university, community, and online store in Southern China. The data were analyzed by Cochran–Mantel–Haenszel on SPSS 26.0 software program. The results showed that self-construal influenced the effect of start temporal landmarks on arousal product preference. Specifically, compared with ordinary temporal landmarks, individuals with interdependent self-construal prefer high arousal products under start temporal landmarks, whereas those with independent self-construal show no significantly different preference for high or low arousal products under the start temporal landmarks. Furthermore, psychological resources play a mediating role. This research extends the theoretical research on self-construal in the field of temporal landmarks and arousal. It also has important practical implications for improving the sales of high arousal products.

## 1. Introduction

Morning should be the most energetic time in the day, and people are eager to replenish their energy throughout the day [1]. Coffee and milk are the most common drinks in the morning, but they give completely different arousal experiences. Coffee can provide an immediate energy boost and sustain vitality [2,3], whereas milk can relax the mind and help the brain calm down [4]. Thus, in the morning, some choose to drink coffee to meet the new day with full energy and others choose milk to begin a new day. This article will use the question of drink choice in the morning and the reasons for it to investigate preferences for high- or low-arousal products.

Arousal is an experience from drowsiness to excitement or a state of readiness for action [5]. Products can be divided into high- and low-arousal products according to the arousal level. High-arousal products can activate individuals’ behavioral motivation, make their heart beat faster, and raise their blood pressure [6]. Low-arousal products suppress motivation, slow the individual’s heart rate, lower blood pressure, and put the person in a state of calmness and relaxation [7]. Temporal landmarks refer to events stored in individual memory, which are different and unique from the moments or events in daily life [8]. Temporal landmarks exert a significant effect on arousal product preferences. Prior research shows that start temporal landmarks divide individuals’ time into three stages (past, present, and future self). People compare these three stages, setting goals to enhance themselves [9,10,11]. Temporal landmarks influence consumer behavior. For example, start temporal landmarks make consumers prefer simple food packages [12], shift their visual attention to the left [13], and boost travel motivation [14]. Recent research suggests that start temporal landmarks, such as morning, Monday, and New Year, can influence consumer preferences for arousal products [12]. However, the existing literature on start temporal landmarks has mainly focused on individuals’ motivation and behavior and less on individual differences and the relationship between self and others (independent vs. interdependent).

Human beings are by nature the sum of all social relations, and they are in a state of conflict between their individual values and the values that society requires them to conform to [15,16]. Self-construal can influence arousal product preferences by temporal processing styles. Self-construal refers to how one judges the relationship between the self and others [17,18]. For the interdependent self-construal, individuals have too many expectations of family and friends in their goals, and many of them live to fulfill the expectations of others and integrate their selves into social relationships [19,20]. China is the representative of the Eastern collectivist culture, which has a culture of integration of self and social environment [21]. In contrast, Western individualism prevails, emphasizing freedom and tolerance of individuality and dissent. Independent self-construal is common in Western cultures; they are less influenced by social relationships and do not impose the complex social relationships on their self-perceptions [22]. Therefore, exploring the differences in arousal preferences of self-construal under temporal landmarks would greatly advance existing research on temporal landmarks.

According to arousal theory [5] and self-construal theory [23], individuals’ self-construal influences their perception of temporal distance. Interdependents tend to perceive future events at proximal temporal distances, believing that they make failure less likely. By contrast, independents tend to perceive future events at a distal temporal distance, believing that goals need a long time to achieve [24]. These differences influence their task motivation and engagement [24,25]. When the demands of the task exceed the resources of the individual coping ability, a threat state occurs; when resources meet the requirements of the task, a challenge response occurs [26]. Psychological resources are those resources that are consumed by the individual to process information and are indispensable for tasks [27]. We propose that self-construal moderates the effect of start temporal landmarks on arousal product preference. Specifically, interdependent self-construal prefers high-arousal products under start temporal landmarks compared to ordinary times, whereas independent self-construal individuals show no significant preference on arousal products. Given that individuals with interdependent or independent self-construal have different perceptions on start temporal landmarks, interdependent individuals act to achieve goals earlier than independent individuals [28]. Interdependent individuals have a stronger demand for psychological resources than independent individuals, so they prefer high-arousal products.

This research provides important contributions in both theoretical and practical aspects. First, this research finds how start temporal landmarks affect individuals’ perceptions of the relationship between self and others, filling the gap of social behavior involving others in research of start temporal landmarks. Second, this study explains the changes in the psychological resource needs of different types of self-construal under the start temporal landmarks, which can enhance the understanding of the internal mechanisms to the consumer behavior of self-construal. Third, our results enrich arousal product literature from the self perspective, shedding light on how the relationship between self and others affects arousal product preferences. From a practical perspective, the findings of the study help managers identify the motivation for people purchasing high-arousal products.

This paper is organized as follows. First, we review the literature related to temporal landmark and self-construal, then predict how self-construal influences individuals’ psychological resource needs and arousal product preferences. Second, we conduct three experiments to verify the effect of self-construal and start temporal landmarks on arousal product preferences. Finally, we discuss the theoretical and practical implications of the findings.

## 2. Literature Review and Research Hypothesis

### 2.1. Start Temporal Landmarks

Temporal landmarks refer to events stored in individual memory, which are different and unique from the moments or events in daily life [8]. Previous studies have divided temporal landmarks into three classifications: public events with significant impact (epidemics), reference points on the calendar (e.g., New Year’s Day and New Year’s Eve), and reference points in personal experience (turning 18) [8].

Temporal landmarks can also divide individuals’ time into three stages (past, present, and future self) [9,29]. On the one hand, individuals tend to assess their past selves more negatively, believing that their past selves are not as good as their present selves and that the past self leads to regret and imperfection. They separate their present selves from their past selves [11]. By contrast, individuals link their present selves to their future selves and tend to be more positive and optimistic in their assessment of their future selves, setting goals to pursue their future successful selves to close the gap between their current poorer state and their future better state [11,30]. Reminding people of a desired future and predicting future temporal landmarks may also increase people’s motivation [31,32]. For example, individuals who are reminded that they are at the beginning of a new semester will study harder than individuals who are not reminded of the temporal landmarks. If the future state was negative, the effect of desired future on motivation appeared [31].

Research on the “fresh start effect” shows that people are more likely to take immediate action to achieve their goals at a significant start temporal landmark [9]. For example, Google searches for diets, gym attendance, and commitment to meeting goals peak in January, but the effect declines month by month [9]. People’s motivation for self-improvement is strong not just at New Year’s, but at the beginning of the week, month, and year or holidays and birthdays [9,11]. Given that these start temporal landmarks remind individuals of the passage of time, they induce people to downgrade past imperfections to the previous period and to plan for their future lives, thereby inspiring ambitious behavior [9,29,33]. In consumer behavior, people with a strong (vs. weak) restart mentality invest in transformative change by changing their environment, including their own consumer choices (e.g., buying a new pair of sunglasses) [34]. They are also more willing to support change programs that help those at risk get a fresh start (e.g., disadvantaged youth and veterans) [34]. Bartels and Rips (2010) found that landmark events in life can change people’s psychological composition, which, in turn, influences intertemporal choice [35]. Scholars have found that individuals stimulated by a start temporal landmark have need for arousal and prefer high-arousal products [12,13]. However, such research mostly focused on individuals’ motivation and behavior under the start temporal landmarks, but there is less research on the cultural and individual differences in the perception of start temporal landmarks. This article will explain the individual differences in the perception of start temporal landmarks.

### 2.2. Start Temporal Landmarks and Psychological Resources

Psychological resources are the resources consumed by the individual to process information and are indispensable for tasks [26]. Psychological resources are one of the conditions for individuals to accomplish their goals and tasks. Individuals cannot act without the support of psychological resources in carrying out various purposeful activities (such as emotional regulation, resistance to temptation, and self-preservation) [26,36]. People need to consume psychological resources in self-control. When the resources are insufficient, people are in a state of mental depletion, and long-term mental depletion will make them feel tired. This exhaustion is not caused by physical fatigue but a psychological and subjective feeling, which is a state caused by the individual’s mental depletion. According to Hobfoll (2002), psychological resources include the things that people value deep down, such as self-esteem, health, peace, and the things that people need to achieve their goals, such as attention and self-confidence. No matter what kind of psychological resources, they can help people achieve career or academic success [27]. Gilbert et al. (2017) classified psychological resources as positive emotions, vitality, and self-efficacy. These psychological resources are closely related to the individual’s ability to accomplish goals and tasks: positive emotions can improve individual judgment and creativity [37,38], sufficient energy allows individuals to think quickly [39], and self-efficacy increases one’s initiative [40,41].

Start temporal landmarks can stimulate individuals’ demand for psychological resources. The normal, ordinary experiences of everyday life can deplete an individual’s psychological resources [10]. These events include a variety of daily decisions (e.g., decisions about time to leave), cognitive tasks (e.g., meetings), and self-control tasks (e.g., executing school schedules) [42]. Start temporal landmarks mean the beginning, and it leads the individual to draw attention to their growth and development to promote the realization of the ideal self [9,11,30]. And the planning of these goals reminds the individual that they need to consume psychological resources to accomplish many tasks [43]. Therefore, when stimulated by the start temporal landmarks, individuals need to acquire sufficient psychological resources to cope with the challenges encountered in the pursuit of their goals.

### 2.3. Self-Construal, Start Temporal Landmarks, and Arousal

Self-construal refers to how one judges the relationship between the self and others. It comprises independent and interdependent self-construal [44]. The relationship between self and others will affect them to view the upcoming events, i.e., the temporal distance from the present to the future. Self-construal affects people’s perception of temporal distance, in turn, affecting their motivation and engagement with the task [24,45].

Under the start temporal landmark, individuals begin to plan for a perfect future, being optimistic that their ideal future selves are attainable [46]. At this point, individuals with independent self-construal emphasize that growth and ambitious goals take time to achieve, and they are likely to view future events as far away from the present in the temporal dimension [24]. Their sensitivity to earnings and success also drives them to focus on the positive, which is more pronounced in the distant (vs. near) future [47]. Therefore, individuals with independent self-construal tend to perceive future events at a distant temporal distance. By contrast, individuals with interdependent self-construal seek safety and security and strive to fulfill their duties and obligations, being more cautious in pursuing their goals and avoiding risk whenever possible [48]. This requires them to pay close attention to their surroundings and to fulfill their obligations promptly. In addition, their sensitivity to loss and failure drives them to focus on the negative and to value time as a scarce resource [47]. Interdependent individuals perceive the time distance between future events and the present as shorter, feel less time, and start taking action to achieve their goals earlier [24]. To accomplish their goals faster, individuals with interdependent self-construal need sufficient psychological resources to provide energy support.

Human activities require increased drive, such as striving to explore new environments and participating in competitive contests [6,49]. An inverted U-shaped relationship was found between arousal and performance in various cognitive tasks [50]. Specifically, each task corresponds to an optimal level of arousal, and activity efficiency decreases when this optimal point is not reached or exceeded [51]. Therefore, individuals tend to maintain an optimal arousal level physically and psychologically. Individuals generally prefer moderate stimulus levels because they induce the optimal arousal level, and they do not like a stimulus that is too low or too high [52]. However, in real life, high-arousal products, such as coffee, roller coasters, and horror movies, help individuals face threats to their self-identity to restore their self-worth [53], so such products are popular.

Kahneman’s (1973) resource limitation theory suggests that each task must be accomplished using psychological resources that can be used together to accomplish several tasks; the total amount of human psychological resources is limited [54]. High-arousal products mobilize and activate physical and mental energy to bring consumers to a state of high arousal, helping motivated consumers to perform their tasks efficiently with minimal physical or mental exertion [55]. As stated, high-arousal products also help individuals face threats to their self-identity to restore their self-worth [53]. For example, proper intake of caffeine (coffee, tea, energy drinks, or soda) can restore vitality, improve concentration, creativity, and problem-solving skills [56].

In summary, start temporal landmarks denote beginnings, and their initiation leads the individual to draw attention to their growth and development to promote the realization of the ideal self [9,29]. Psychological resources can provide energy support for the consumer’s mental activity or behavior. In addition, the normal, mundane experiences of everyday life can deplete one’s ability to resist temptation [10,57]. Interdependent individuals find that their future perfect selves are closer to their present selves, serving as motivation to achieve their goals. Compared with independent self-construal individuals, interdependent self-construal individuals have a stronger need for psychological resources, especially after being stimulated by start temporal landmarks. Therefore, the following hypotheses are proposed in this paper:

**H1.**  *Compared with ordinary times, interdependent consumers prefer high-arousal products under start temporal landmarks, whereas this effect is not shown among independent consumers.*

**H2.**  *Compared with ordinary times, start temporal landmarks lead to a desire for psychological resources among interdependent consumers, leading to a preference for high-arousal products, whereas this effect is not shown among independent consumers.*

## 3. Overview of Studies

We will conduct three experiments to verify the hypothesis. Study 1 examined the effect of temporal landmark and self-construal on high-arousal product preference. Study 2 adopted real temporal landmark and arousal music, verifying again the effect of start temporal landmark and self-construal on the preference of high-arousal products. Study 3 measured participants’ psychological resource needs at the start temporal landmark, using a moderated mediation method to verify the mediating role of psychological resources. To enhance the validity and robustness of the experiment, we used a combination of the student (Study 2) and nonstudent samples (Study 1 and 3). We conducted two laboratory experiments (Study 1 and 2) and an online experiment (Study 3). We also used different time points to manipulate participants’ perceptions of beginning. Also, in these studies, we measured high-arousal product preference with different products. The details are shown in the table below (see Table 1).

## 4. Study 1

### 4.1. Participants and Design

Study 1 initially verified H1 that interdependent consumers prefer products with high arousal levels under the start temporal landmarks compared to ordinary time. The study consisted of a 2 (temporal landmark: start group vs. control group) × 2 (self-construal: independence vs. interdependence) between subjects. We asked participants to read a story to manipulate self-construal (independence vs. interdependence). Then, we manipulated participants’ perception of the start temporal landmarks through contextualized pictures and directly asked participants to choose between high- and low-arousal products (see Table 2). Residents of South China were recruited to participate in Study 1. The residents are representative because they are more diverse in terms of age, income, and social experience. A total of 136 residents of a community in Southern China (87 females, M_age_ = 25.08, SD = 4.66) participated in this study. All the residents received a small cash payment of CNY 6 at the end of the study. We chose a two-way ANOVA of SPSS 26.0 to analyze the sample size and, when the number of groups was 4, the degree of freedom was 1, the effect amount (f) was 0.25, and the significance level was 0.05, the power value of the sample size of 136 was 0.99, exceeding the base level of 0.80.

### 4.2. Materials and Methods

Participants were randomly assigned to read one of the following scenarios (independence vs. interdependence). All participants were asked to read a story [58]: “Sostoras, a warrior, helped Sargon I to take over the world, and the king rewarded him with a territory. Some years later, the country was facing a new war. The king asked Sostoras to nominate a man to command the army. After careful consideration, Sostoras decided to nominate a general named Tiglath”. The reasons for the independence and interdependence groups’ recommendations are different.

**[independence]** Sostoras recommended Tiglath because he considered that Tiglath was his fellow and that the king would reward himself for recommending such a talented man and that Tiglath would be grateful to him for it, which would help to consolidate his power and increase his prestige.

**[interdependence]** Sostoras recommended Tiglath because he was a member of his family and recommending him would help increase his family’s power and prestige and would show his family members that he was loyal to his family.

To ensure that participants read the material carefully, they were asked to answer a question “Sostoras was”, and the correct answer is warrior. The data of participants who answered incorrectly would not be analyzed. Meanwhile, to ensure the manipulation in priming independent and interdependent self-construal, we used four items to measure the participants’ self-focus and others-focus on the extent of the others [48]. “Please describe the extent to which (1) your thoughts about the message were focused on just yourself, (2) your thoughts were focused on just you, (3) your thoughts about the message were focused on you and your family, and (4) your thoughts were focused on you and your family”. The first two items were used to measure the participants’ self-focus (α = 0.846), and the remaining two items were used to measure the participants’ others-focus (α = 0.832).

Next, the participants looked at two pictures and described how they felt afterward. Drawing on previous research, we asked participants to describe how these two images manipulated their perception of the start temporal landmarks [59]. In the start condition, participants saw two scenes taken at 8:00 a.m. In the control condition, participants saw two scenes taken at a park without a specific time. In the start condition, the participants were asked to imagine that they are at the time when the picture is presented, and the participants in the control condition were asked to imagine that they are at the place where the picture is presented. The participants in both groups were asked to imagine how they feel and what they would do at that time [13,34]. After viewing the pictures (see Appendix A), they were asked to describe exactly how they felt at the moment around the following question and answer the manipulation of the start temporal landmark to check “to what extent does the picture information make me feel that the day has just begun?” (1 = strongly disagree, 7 = strongly agree; α = 0.942).

Finally, we informed the participants that we wanted to know their drink taste preferences for market research. We asked them to choose one of two drinks according to the instructions in a flyer. According to Chen (2019), we manipulated the arousal level of the product using AdWords. Both drinks have the same brand, same price, and similar packaging style. High-arousal drinks focus on “Invigorating the mind, awakening the body, and replenishing energy”. Low-arousal drinks focus on “Soothing the nerves, relaxing the body, and enjoying peace”. (see Appendix B). Participants needed to choose between the two drinks according to their feelings: “If you were to buy one of the two drinks, which one would you choose?” Finally, we collected demographic variables, such as age and gender of the participants, and gave them certain remuneration. Data were analyzed by the statistical programs SPSS.

### 4.3. Results

*Manipulation checks.* Three participants failed to pass the attention question, and their data were deleted in subsequent analyses. There were 133 valid data (87 females, Mage = 25.08, SD = 4.66). The experimental data were analyzed by ANOVA. The extent of perceived beginning was higher in the start temporal landmark condition (M = 5.88, SD = 1.46) than in the control condition (M = 3.69, SD = 1.62; F (1, 131) = 62.640, *p* < 0.001). We also analyzed participants’ extent of self-focus and others-focus separately. The extent of self-focus was higher for independent participants (M_independent_ = 5.41, SD = 1.07) than those for interdependent participants (M_interdependent_ = 4.53, SD = 1.09, F (1, 131) = 16.356, *p* < 0.001). Participants with interdependent self-construal had a higher extent of other-focus (M_interdependent_ = 5.25, SD = 1.14) than those with independent self-construal (M_independent_ = 4.51, SD = 3.37, F (1, 131) = 16.356, *p* < 0.001; see Table 3). This demonstrates the success of the start temporal landmark and self-construal manipulation.

**Figure 1:**
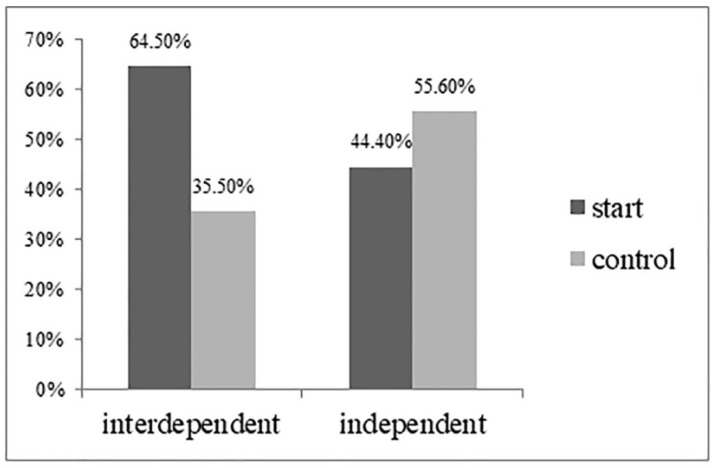
High-arousal drink preferences (Study 1).

*High-arousal product preference.* According to the activated self-construal (interdependence vs. independence), we compare the proportion of people who choose high-arousal products with the proportion of people who choose low arousal products. Cochran–Mantel–Haenszel results showed a significant interaction between temporal landmark and self-construal (Wald χ^2^ = 9.00, *p* = 0.003). As predicted, when interdependent self-construal was activated, the start temporal landmarks made more people choose high-arousal products (64.5%) than the control condition (35.5%; Wald χ^2^ = 9.092, *p* < 0.05). When independent self-construal was activated, no significant difference was observed in the arousal level of the product with the start temporal landmarks (44.4% vs. 55.6%, Wald χ^2^ = 2.099, *p* = 0.15) (see Figure 1), consistent with H1.

### 4.4. Discussion

Study 1 showed that interdependent individuals are more likely to choose high-arousal products at the start temporal landmarks than at ordinary times. When activating independent self-construal, whether the start temporal landmarks were used, no significant preference was observed between high-arousal and low-arousal products. However, this study only used a simple picture description to stimulate participants to initiate the start temporal landmarks. The simple picture description does not represent the participants’ real feelings about the beginning. Whether real-time landmarks affect the preference for high arousal of self-construal is not yet known, a problem solved in Study 2. In addition, drinks can be consumed directly and can physically regulate arousal levels directly. People may be concerned about the impact of some functional drinks on their health, whereas music can regulate arousal levels without any contact with people. Thus, Study 2 used arousal music to exclude the effect of people’s health concerns on the results of the experiment.

## 5. Study 2

### 5.1. Participants and Design

Different from Study 1, which used pictures to manipulate the start temporal landmark, Study 2 adopted real-time landmarks to manipulate participants’ perception of the temporal landmark. This approach causes participants to be truly stimulated by the start temporal landmark, further exploring the effect of self-construal on arousal product preference under the start temporal landmark and increasing the robustness of this effect. Study 2 was conducted in the laboratory, with the participation of 486 students (274 females, M_age_ = 26.27, SD = 7.56) from a university in South China. Each student received course credit at the end of the experiment. The study consisted of a 2 (temporal landmark: start vs. control) × 2 (self-construal: independence vs. interdependence) between subjects (see Table 4). We chose a two-way ANOVA of SPSS 26.0 to analyze the sample size and, when the number of groups was 4, the degree of freedom was 1, the effect amount (f) was 0.25, and the significance level was 0.05, the power value of the sample size of 486 was 0.88, exceeding the base level of 0.80.

### 5.2. Materials and Methods

This experiment was conducted on 19 April 2022. The start temporal landmark was manipulated by informing the participants that the day of the experiment was Monday. The participants were required to record the date of the experiment. In the condition of start temporal landmark, the instructional phrase was “Today is Monday, signaling the beginning of the week”. In the control condition, the instructional phrase was “Today is the 19th of the month, an ordinary day”. To ensure that participants read the material carefully, they were asked to answer “the day of the experiment was”, and the correct answer is “19 April” or “Monday”. The data of participants who answered incorrectly would not be analyzed. Subsequently, participants were asked whether they agreed that “today means a new beginning” and “today is a special day” (1 = strongly disagree, 7 = strongly agree, α = 0.735) as a manipulation check of the start temporal landmark. We measured the participants’ time orientation to verify and exclude its effect on the results. Participants were asked “To what extent does today remind me of the past or future week (1 = the past week, 7 = the future week)”. Next, in the independent condition, participants were asked to answer two questions in two minutes, “What makes you different compared to others around you?” and “What are your expectations of yourself?” In the interdependent condition, participants were asked to answer the questions “What are the similarities between you and the people around you?” and “What do your family and friends expect of you?” To test the validity of this manipulation, participants were asked to complete the self-construal scale (α = 0.835) [60].

Finally, we informed the participants that we wanted to know their musical style preference. The participants were shown two pieces of Beethoven’s music. According to Chen et al. (2019), the Dance Symphony is high-arousal music, which is cheerful, bright, positive, full of passion, and exciting; the Garden Symphony is low-arousal music, which is delicate, simple, and peaceful, allowing people to feel the beauty of nature (see Appendix C). The participants chose which symphony they liked the most (0 = “Garden Symphony”, 1 = “Dance Symphony”). Finally, we collected demographic variables, such as age and gender, of the participants. Data were analyzed by the statistical program SPSS.

### 5.3. Results

*Manipulation checks.* Six participants failed to pass the attention question and five participants’ data were missing. These data were deleted in the subsequent analysis, leaving 475 valid data. ANOVA revealed that the extent of perceived beginning was significantly higher in the condition of start temporal landmark (M = 4.79, SD = 1.82) than in the control condition (M = 3.77, SD = 1.73; F (1, 473) = 38.98, *p* < 0.001). We analyzed the independent and interdependent self-awareness items separately, and participants with independent self-construal scored higher on the independent self-awareness index (M_independent_ = 5.52, SD = 0.88) than those with interdependent self-construal (M_interdependent_ = 4.86, SD = 0.74, F(1,475) = 51.89, *p* < 0.001). Participants with interdependent self-construal scored higher on the interdependent self-awareness index (M_interdependent_ = 5.61, SD = 1.05) than did those with independent self-construal (M_independent_ = 5.19, SD = 0.84, F (1, 475) = 20.72, *p* < 0.001; see Table 5). This demonstrates the success of the start temporal landmark and self-construal manipulation.

**Figure 2:**
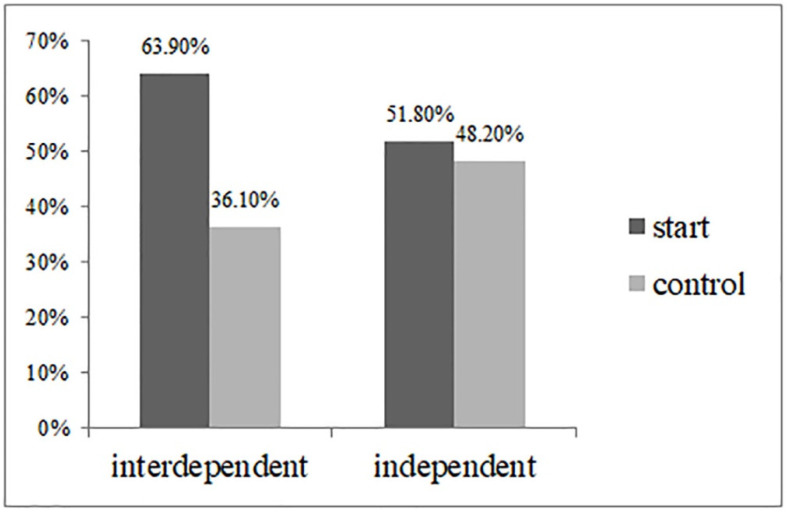
High-arousal music preferences (Study 2).

*High-arousal product preference.* We conducted a Cochran–Mantel–Haenszel test with self-construal, temporal landmark, and the interaction term as independent variables and high-arousal product preference as a dependent measure. The results showed that the interaction between temporal landmark and self-construal was significant, as predicted (Wald χ^2^ = 6.429, *p* < 0.001). As shown in Figure 2, in the start temporal landmark condition, participants in the interdependent self-construal condition were more likely to choose high arousal products (63.9%) than the control condition (36.1%; χ^2^ (1) = 8.94, *p* < 0.05). By contrast, in the independent self-construal condition, no significant differences were found in high-arousal product preferences in the condition of the start temporal landmark and control condition (51.8% vs. 48.2%; χ^2^ (1) = 18.60, *p* = 0.64; see Figure 2). The results support H1.

### 5.4. Discussion

Study 2 allowed participants to realistically perceive the start temporal landmark. Study 2 further verified the effect of self-construal on arousal product preference at the start temporal landmark (H1). However, 8:00 a.m. and Monday are common calendar landmarks. Study 1 and 2 examined the high-arousal product preferences of self-construal at calendar landmarks with beginnings, that is, by manipulating the start temporal landmark with 8:00 a.m. and Monday, respectively. Do meaningful time points (e.g., personal experience) also affect the relationship between self-construal and arousal product preferences at the start temporal landmark? Study 3 will test the internal validity of the experiment using special meaningful time points to manipulate the start temporal landmark. Study 3 further verified psychological mechanisms on the relationship between self-construal and arousal product preference at the start temporal landmark.

## 6. Study 3

### 6.1. Participants and Design

Study 3 verifies the external validity of results using special meaningful time points to manipulate the start temporal landmark. This study further demonstrates that interdependent individuals’ preference for high-arousal products is driven by a desire for psychological resources. We co-operated with an online store and conducted a week-long study. We designed advertisements for two drinks and sent advertising messages to 536 customers. All customers who completed the survey received a CNY 10 no-threshold coupon. The study consisted of a 2 (temporal landmark: start vs. control) × 2 (self-construal: independence vs. interdependence) between subjects (see Table 6). We chose a two-way ANOVA of SPSS 26.0 to analyze the sample size and, when the number of groups was 4, the degree of freedom was 1, the effect amount (f) was 0.25, and the significance level was 0.05, the power value of the sample size of 536 was 0.90, exceeding the base level of 0.80.

### 6.2. Materials and Methods

Participants were randomly assigned to four experimental conditions. Before they saw the advertising message, participants were asked to imagine a tennis match as a solo player or as part of a team. Participants immediately completed a self-construal survey as a manipulation check. Referencing Aaker and Lee (2001), we used four items to measure participants’ self-focus and others-focus: “My thoughts are focused on myself”, “I focus more on myself”, “My thoughts are focused on me and my teammates”, and “I focus more on me and my teammates”. Then, in the start condition, the instruction was “It’s the first day of the semester. You’re playing in a tennis tournament and you’ve made it to the finals”. In the control condition, the instruction was “Today is the 117th day of the semester. You are participating in the tennis tournament and have reached the finals”. To ensure that participants read the material carefully, they were asked to answer “the day of the tennis match was”, and the correct answer is “the 1st day of the semester” or “the 117th day of the semester”. The data of participants who answered incorrectly would not be analyzed. Subsequently, participants were asked whether they agreed that “today means a new beginning” and “today is a special day” (1 = “strongly disagree”, 7 = “strongly agree”; α = 0.879) as a manipulation check of the start temporal landmark. Next, the extent to which participants need psychological resources was measured by adopting Kim et al.’s (2018) scale of psychological resources, which asks participants to what extent they want to become awake, energetic, confident, and pleasant at this time (1 = “strongly disagree”, 7 = “strongly agree”; α = 0.872).

Finally, we informed the participants that we wanted to know their drink taste preferences. Participants read that high-arousal drinks focus on “Invigorating the mind, awakening the body and replenishing energy” and that low-arousal drinks focus on “Soothing the nerves, relaxing the body, and enjoying peace”. (see Appendix B). Participants were required to choose between the two drinks according to their feelings: “If you were to buy one of the two drinks, which one would you choose?” We also collected demographic variables, such as age and gender, of the subjects. Data were analyzed by the statistical program SPSS.

### 6.3. Results

*Manipulation checks.* Five participants failed to pass the attention question and three participants’ data were missing. These data were deleted in the subsequent analysis, leaving 475 valid data. ANOVA revealed that the extent of perceived beginning was higher in the condition of start temporal landmark (M = 5.06, SD = 1.33) than in the control condition (M = 4.33, SD = 1.49; F (1, 526) = 34.784, *p* < 0.001). We also separately analyzed participants’ extent of self-focus and others-focus. The extent of self-focus was higher for participants with independent self-construal (M_independent_ = 4.91, SD = 1.16) than for those with interdependent self-construal (M_interdependent_ = 4.45, SD = 1.34, F (1, 526) = 18.175, *p* < 0.001). Participants with interdependent self-construal had a higher extent of others-focus (M_interdependent_ = 5.28, SD = 1.09) than those with independent self-construal (M_independent_ = 4.93, SD = 1.18, F(1, 526) = 12.518, *p* < 0.001; see Table 7). This demonstrates the successful manipulation of the start temporal landmark and self-construal.

**Figure 3:**
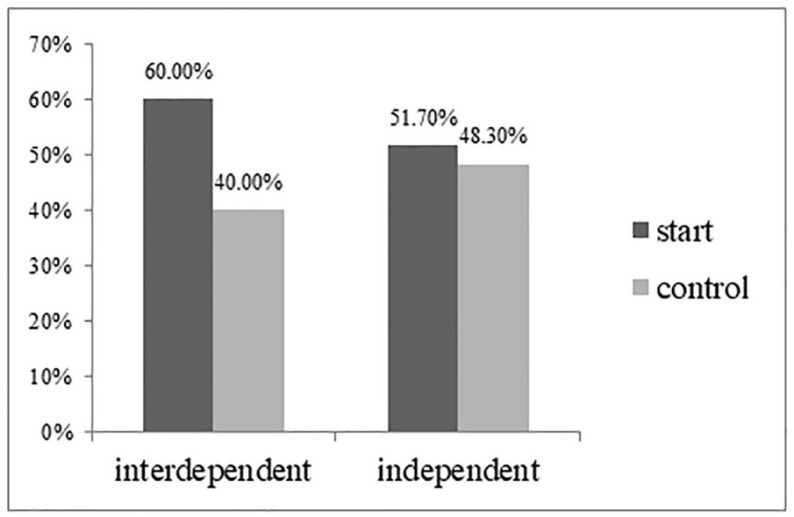
High-arousal drink preferences (Study 3).

*High-arousal product preference.* We conducted a Cochran–Mantel–Haenszel test with self-construal, temporal landmark, and the interaction term as the independent variables and high-arousal product preference as the dependent variable. As predicted, under the start temporal landmark, participants in the interdependent self-construal condition were more likely to choose high-arousal products (60%) than those in the control condition (40%; χ^2^ (1) = 6.974, *p* < 0.05). By contrast, in the independent self-construal condition, no significant difference was found in high-arousal product preferences in the condition of the start temporal landmark and control condition (51.7% vs. 48.3%; χ^2^ (1) = 0.245, *p* = 0.62) (see Figure 3). The results support H1.

*Psychological resources.* An ANOVA on psychological resources revealed a significant interaction effect (F (1, 524) = 27.459, *p* < 0.001). Specifically, among the interdependent participants, those at the start temporal landmark showed a stronger need for psychological resources (M _start_ = 5.51, SD = 1.03) than did those at ordinary times (M _control_ = 4.86, SD = 0.69; F (1, 524) = 40.944, *p* < 0.001). This effect was attenuated among the independent participants (M _start_ = 4.79, SD = 0.77 vs. M _control_ = 4.89, SD = 0.77; F (1, 524) = 1.024, *p* > 0.05; see Table 7).

**Figure 4:**
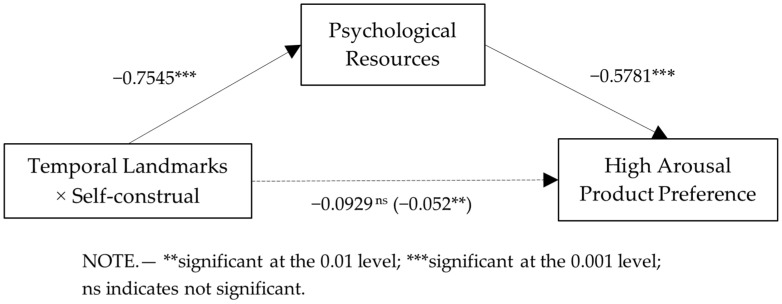
Psychological resource as a mediator (Study 3).

*Moderated mediation analysis.* First, we regressed the arousal product preferences on start temporal landmark, self-construal, and their interaction in a binary logistic regression. This analysis only resulted in a significant interaction effect (β = −0.0929, *p* < 0.01). Second, we regressed psychological resources on start temporal landmark, self-construal, and their interaction, which revealed a significant interaction effect (β = −0.7545, *p* < 0.001). Finally, we regressed the arousal product preferences on start temporal landmark, self-construal, start temporal landmark × self-construal, psychological resources, and psychological resources × self-construal. The results revealed that the main effect of psychological resources remained significant (β = 0.5781, *p* < 0.001). Third, we tested whether psychological resources mediated the effect of the start temporal landmark on the arousal product preferences and whether self-construal moderated the path from the start temporal landmark to psychological resources. A moderated mediation analysis (Hayes 2017, PROCESS Model 7 with 5000 bootstrap samples) with psychological resources as the mediator and self-construal as the moderator revealed that, in the interdependent condition, the indirect effect of the start temporal landmark on arousal product preferences via psychological resources was significant (b = −0.3766, SE = 0.1006, 95% CI = [−0.5989, −0.2072]). However, in the independent conditions, this indirect effect was not significant (b = 0.0596, SE = 0.0573, 95% CI = [−0.0464, 0.1823]) (see Figure 4). The results support H2.

### 6.4. Discussion

Study 3 further validated the finding that the interdependent individuals preferred the high-arousal product more at the start temporal landmark than at ordinary times; this effect was not shown among the independent individuals. This study further demonstrates that interdependent individuals’ preference for high-arousal products is driven by psychological resources. The start temporal landmark for participant initiation in this study is special meaningful times different from Study 1 and Study 2, enhancing the external validity of the results.

## 7. General Discussion

Based on self-construal theory, this research extended self-construal to the effect of start temporal landmarks on arousal product preferences. Consistent with existing relevant research, individuals prefer high-arousal products under the start temporal landmark, but this effect only appears in interdependent individuals. We used three experiments to test the assumption, and the experimental results were all consistent with the assumption. The three studies used different start temporal landmarks to validate the hypothesis, namely, 8:00 a.m. in Study 1 (contextualized pictures), Monday in Study 2 (real time), and the first day of the new semester in Study 3 (meaningful temporal landmarks). Through manipulating the activation context self-construal and the start temporal landmark, Study 1 examined whether start temporal landmark influenced the preference for high arousal products among interdependents and independents. Specifically, interdependent self-construal preferred high-arousal products more under the start temporal landmark than ordinary times, whereas independent individuals did not show significant preference on high- and low-arousal products, regardless of whether they were at the start temporal landmark (H1). Study 2 manipulated the start temporal landmark in real time, further verifying this effect (H1). Study 3 used meaningful landmarks (the first day of the new semester) to stimulate participants to initiate the start temporal landmark and using a guidance word to manipulate the self-construal, further verifying this effect (H1). Study 3 further analyzed the mediating role of psychological resources. Start temporal landmarks led to a desire for psychological resources among interdependent consumers, leading to a preference for high-arousal products (H2).

### 7.1. Theoretical Contribution

First, this paper expands the literature on “fresh start” effects involving visual cues such that fresh starts motivate individuals’ aspirational behavior [9], increase retirement savings immediately [61], and ethical behavior [10]. The existing literature on start temporal landmarks mainly focused on self-perceptions and motivations [8,62,63,64] and less on the relationship between the self and others. This paper investigates the relationship between self-construal and start temporal landmarks, filling the gaps in existing research on the effects of start temporal landmarks on consumer behavior. The present study confirms that self-construal has different perceptions of temporal landmarks. Specifically, faced with fresh starts, independent individuals perceive a far temporal distance when considering future events, and the future self is more distant from the present self (i.e., the goal completion is perceived as taking time to complete). However, interdependent individuals perceive a closer temporal distance as distant, and the future self is closer to the present self (i.e., the time resources are perceived as scarce).

Second, prior research shows that psychological resources are related to the individual’s ability to accomplish goals and tasks [65,66], and individuals need psychological resources to accomplish goals and tasks to improve energy support [27,67]. This research enriches the study of psychological resources and verifies that self-construal has different demands for psychological resources under the start temporal landmarks. Interdependent individuals have a stronger need for psychological resources than do independent individuals, especially under the start temporal landmarks. This study also demonstrates that psychological resources are factors that influence arousal product preferences, enriching the research related to psychological resources. An individual’s desire for psychological resources causes them to spontaneously engage in behaviors that can satisfy this desire, such as consuming high-arousal products.

Finally, prior research shows that individuals show a preference for high arousal products in situations such as emotional regulation, distraction, and threats to their self-identity [6,26,68]. The current research incorporates self-construal into the scope of exploration, further expanding the study of self-construal in arousal. Our results show that self-construal also has different arousal preferences under the start temporal landmarks. Interdependent individuals will act to achieve their goals earlier than independent individuals [25,28]. They will tend to keep their energy levels high at the start temporal landmarks and try to accomplish their goals with the least amount of energy consumption. Thus, compared with independent individuals, interdependent individuals prefer high-arousal products under start temporal landmarks.

### 7.2. Managerial Implications

By exploring the relationship between start temporal landmarks and high arousal products, this study helps managers clarify people’s motivation to purchase high-arousal products. They can conduct precise marketing according to different periods (e.g., morning vs. noon vs. evening and beginning of the month vs. end of the month) and different consumer types (interdependent vs. independent). The practical significance of this study is reflected in the following two aspects.

First, the difference between high-arousal products and low-arousal products is the arousal experience, an experience that changes from drowsiness to excitement. High-arousal products can activate individuals’ behavioral motivation and can satisfy the need for psychological resources. People are more likely to take immediate action to achieve their goals at a significant start temporal landmark. Their activities require increased drive. Thus, when promoting high-arousal products, such as coffee, tea, and energy drinks, marketers can add some descriptions with the meaning of “beginning”, for example, the first day of a new semester, Monday, etc. Low arousal products suppress motivation, slow the individual’s heart rate, lower blood pressure, and put the person in a state of calmness and relaxation. Therefore, when promoting juice, milk, lavender-scented products, and other low-arousal products, marketers should avoid selling or promoting them at the time of “start” and position them for use when consumers are tired and exhausted and require time to rest.

Second, those with interdependent self-construal have a defensive need to avoid failure, they seek safety and security, and strive to fulfill their duties and obligations, being more cautious in pursuing their goals and avoiding risk whenever possible. The experimental section focuses on different manipulation methods to initiate different types of self-construal in the short term. For example, “we” represents the interdependent self-construal and “I” represents the independent self-construal. The major difference between “we” and “I” is whether self-behavior and attitudes depend on emotions and thoughts in relation to others. “We” emphasizes the social network in which consumers live. Their consumption choices are determined by the thoughts and feelings they perceive in relation to “we” (i.e., external information). “I” is an attitude and behavior determined by the inner thoughts and feelings of the self and is not influenced by the emotional messages from external information (advertisers and businesses). Therefore, the use of “we” in advertising slogans can enhance consumers’ arousal level more than “I”, and this effect can encourage consumers to buy high-arousal products.

### 7.3. Limitations and Future Research

The present study is not without limitations, and its shortcomings are reflected in the following areas. First, regarding psychological resources, we measured self-reported psychological resource needs, but differences exist between physically present psychological resources and perceived psychological resources [69]. These differences may affect the validity of the study findings. Therefore, future studies could measure physically present psychological resources in other ways, such as heart rate and skin conductance tests [70]. In addition, this study only investigated the start temporal landmarks, which includes both the start temporal landmarks and the end temporal landmarks, and future studies can further explore the effect of the end temporal landmarks, such as consumer persuasion [71], debt stress and luxury consumption [72], firm financial performance [73], and online media synergy [74].

This study also focused on the effect of high-arousal products on psychological resources. Studying the reasons why consumers buy low-arousal products is also important. Previous studies have shown that mental resources can also be replenished by rest and the scents of strawberry and lavender and that low-arousal products have a soothing and relaxing effect on the body [75], helping individuals recover depleted mental resources from fatigue and are found everywhere in daily life. Love of nature also affects consumer behavior [76]. Low-arousal products, such as Beethoven’s Garden Symphony, are often related to nature. This attribute may be a reason why consumers buy low-arousal products.

Finally, individuals unconsciously rely on emotions for decision making when faced with different particular situations [77,78,79,80]. Does this influence amplify when the temporal landmark is relative to other ordinary time points? Independent individuals also pursue uniqueness and freedom and have higher uniqueness needs. Individuals with high demand for uniqueness prefer unique products [81], and high-arousal products can increase self-worth [68]. Independent individuals may choose high-arousal products to satisfy their demand for uniqueness under start temporal landmarks. Locus of control and relationship orientation [82,83] could influence consumers’ purchase decisions. Thus, future research can further investigate other boundary conditions.

## Figures and Tables

**Table 1:** Overview of studies.

Study	Sample	Manipulation	Main Purposes
Study 1	Community	Temporal landmarks: two scenes taken at 8:00 a.m. (vs. a park) Self-construal: a story about family benefit (vs. self-benefit) Arousal products: drinks	Examining initially H1 that interdependent consumers prefer high-arousal products under the start temporal landmarks than ordinary time
Study 2	University	Temporal landmarks: April 19 vs. Monday Self-construal: Similarities (vs. differences) with others Arousal products: music	Further examining the effect of self-construal on arousal product preference at the start temporal landmark (H1)
Study 3	Online store	Temporal landmarks: the first (vs. 117th) day of the semester Self-construal: participate in a tennis match as part of a team (vs. a solo player) Arousal products: drinks	Examining the mediating role of psychological resources (H2)

**Table 2:** Participants and procedure in Study 1.

Study 1			
N = 136, 87 Females, M_age_ = 25.08 Years, SD = 4.66, Community in Southern China			
	Step 1	Step 2	Step 3
Start—Interdependent (N = 34)	“Read a story to priming interdependent self-construal”	“Saw two scenes taken at 8:00 a.m.”	“Choose high arousal drinks or low arousal drinks”
Control—Interdependent (N = 34)		“Saw two scenes taken at a park without a specific time”	
Start—Independent (N = 34)	“Read a story to priming independent self-construal”	“Saw two scenes taken at 8:00 a.m.”	
Control—Independent (N = 34)		“Saw two scenes taken at a park without a specific time”	

**Table 3:** Statistics descriptive results in Study 1.

Study 1				
N = 133, 87 Females, M_age_ = 25.08 Years, SD = 4.66, Community in Southern China				
	Interdependent		Independent	
	Start	Control	Start	Control
Perceived beginning	5.87 (1.55)	3.28 (1.52)	5.91 (1.38)	4.07 (1.55)
Self-focus	4.50 (1.27)	4.55 (0.95)	5.91 (0.87)	5.14 (1.08)
Others-focus	5.68 (1.11)	4.91 (1.06)	4.75 (1.12)	4.38 (0.83)
High-arousal drinks preference	64.5%	35.5%	44.4%	55.6%

Notes: statistics in parentheses are standard deviations.

**Table 4:** Participants and procedure in Study 2.

Study 2			
N = 486, 274 Females, M_age_ = 26.27, SD = 7.56, University in Southern China			
	Step 1	Step 2	Step 3
Start—Interdependent (N = 121)	“Today is Monday, signaling the beginning of the week”.	“What are the similarities between you and the people around you?” and “What do your family and friends expect of you?”	“Choose high arousal music or low arousal music”.
Control—Interdependent (N = 121)	“Today is the 19th of the month, an ordinary day”.		
Start—Independent (N = 122)	“Today is Monday, signaling the beginning of the week”.	“What makes you different compared to others around you?” and “What are your expectations of yourself?”	
Control—Independent (N = 122)	“Today is the 19th of the month, an ordinary day”.		

**Table 5:** Statistics descriptive results in Study 2.

Study 2				
N = 475, 272 Females, M_age_ = 26.07 Years, SD = 7.59, University in Southern China				
	Interdependent		Independent	
	Start	Control	Start	Control
Perceived beginning	4.81 (1.82)	3.71 (1.62)	4.77 (1.82)	3.84 (1.84)
Independent self-awareness	4.94 (0.71)	4.78 (0.78)	5.56 (0.86)	5.49 (0.91)
Interdependent self-awareness	5.72 (0.94)	5.51 (1.15)	5.23 (0.81)	5.16 (0.81)
High-arousal music preference	63.9%	36.1%	51.8%	48.2%

Notes: statistics in parentheses are standard deviations.

**Table 6:** Participants and procedure in Study 3.

Study 3			
N = 536, 336 Females, M_age_ = 34.09 Years, SD = 9.21, Online Store in China			
	Step 1	Step 2	Step 3
Start—Interdependent (N = 134)	“It’s the first day of the semester. You’re playing in a tennis tournament and you’ve made it to the finals”.	“Imagine a tennis match as part of a team”	“Choose high arousal drinks or low arousal drinks”
Control—Interdependent (N = 134)	“Today is the 117th day of the semester. You are participating in the tennis tournament and have reached the finals”.		
Start—Independent (N = 134)	“It’s the first day of the semester. You’re playing in a tennis tournament and you’ve made it to the finals”.	“Imagine a tennis match as a solo player”	
Control—Independent (N = 134)	“Today is the 117th day of the semester. You are participating in the tennis tournament and have reached the finals”.		

**Table 7:** Statistics descriptive results in Study 3.

Study 3				
N = 528, 339 Females, M_age_ = 34.19 Years, SD = 9.21, Online Store in China				
	Interdependent		Independent	
	Start	Control	Start	Control
Perceived beginning	5.15 (1.30)	4.31 (1.42)	4.96 (1.34)	4.35 (1.56)
Self-focus	4.62 (1.30)	4.27 (1.37)	4.90 (1.17)	5.93 (1.16)
Others-focus	5.23 (1.13)	5.33 (1.04)	4.89 (1.20)	4.98 (1.15)
High-arousal drinks preference	60%	40%	51.7%	48.3%
Psychological resources	5.51 (1.03)	4.86 (0.69)	4.79 (0.77)	4.89 (0.77)
	95% CI = [−0.5989, −0.2072]		95% CI = [−0.0464, 0.1823]	

Notes: statistics in parentheses are standard deviations.

**Figure A1:**
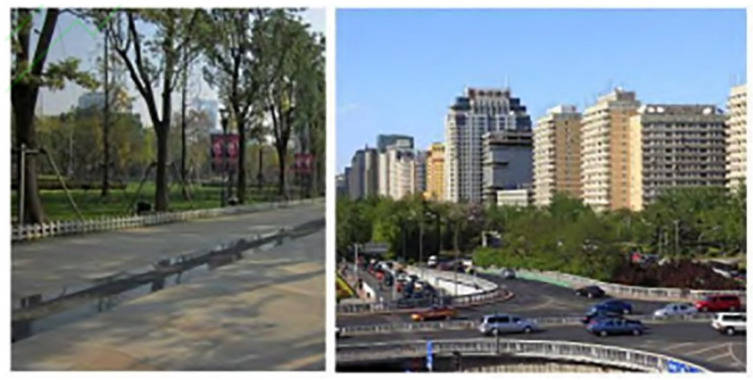
Urban streetscape taken at 8:00 a.m.

## Data Availability

The data presented in this study are available on request from the corresponding author. The data are not publicly available due to the need to maintain the confidentiality of study participants.

## Appendix A

Scenes Stimuli Used in Study 1.

## Appendix B

**Table A1:** Arousal drinks stimuli used in Study 1 and Study 3.

High-Arousal Drink	Low-Arousal Drink

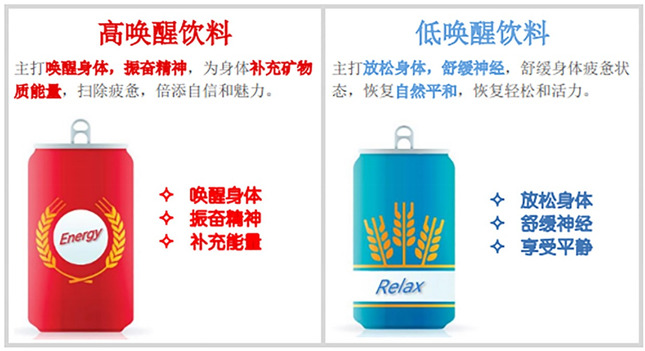

High-arousal drink focuses on awakening the body, invigorating the spirit, replenishing the body with mineral energy, eliminating fatigue, and adding confidence and charm.	Low-arousal drink focuses on relaxing the body, soothing the nerves, relieving the body of its tired state, restoring natural peace, relaxation, and vitality.

## Appendix C

**Table A2:** Arousal music stimuli used in Study 2.

High-Arousal Music	Low-Arousal Music

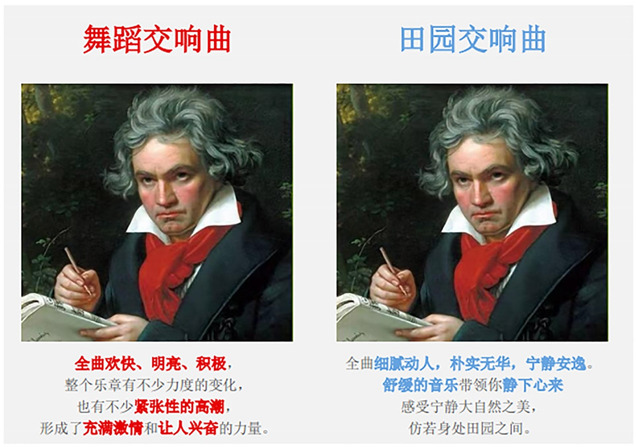

The song is cheerful, bright and positive. There are many changes of intensity throughout the movement. There are also a number of tense climaxes. The movement is full of passionate and exciting power.	The song is delicate and moving, simple and unpretentious, peaceful and restful. The soothing music leads you to quiet your mind, feel the beauty of nature and tranquility. It is as if you are in an idyllic place.
